# Bone health in children with severe cerebral palsy

**DOI:** 10.3389/fped.2023.1264111

**Published:** 2023-11-29

**Authors:** Vincent Barbier, Vincent Goeb, Richard Gouron, Severine Fritot, Romuald Mentaverri, Céline Klein

**Affiliations:** ^1^ Department of Paediatric Physical Medicine and Rehabilitation, Amiens University Hospital and Jules Verne University of Picardie, Amiens, France; ^2^ Three Valleys Rehabilitation Center, Victor Pauchet Group, Corbie, France; ^3^ Department of Paediatric Physical Medicine and Rehabilitation, Corbie Hospital, Corbie, France; ^4^ MP3CV-UR-UPJV 7517, CURS, Amiens University Hospital, Jules Verne University of Picardie, Amiens, France; ^5^ Department of Rheumatology, Amiens University Hospital and Jules Verne University of Picardie, Amiens, France; ^6^ Department of Paediatric Orthopaedic Surgery, Amiens University Hospital and Jules Verne University of Picardie, Amiens, France; ^7^ Department of Biochemistry and Endocrine Biology, Amiens University Hospital, Jules Verne University of Picardie, Amiens, France

**Keywords:** cerebral palsy, bone, low bone mineral density, osteoporosis, bone fragility

## Abstract

**Aim:**

To describe bone health and associated factors in children with severe cerebral palsy.

**Method:**

In a retrospective, single-centre study, we performed a comprehensive bone evaluation (including clinical, densitometric and bone biomarker assessments) of children with severe cerebral palsy.

**Results:**

None of the 19 included children had a normal BMC^TBLH^ Z score, and only one had a BMD^TBLH^ Z score greater than −2. Six children had a BMD^LS^ Z score greater than −2. The bone biomarker data were suggestive of excessive bone remodelling. Levels of bone remodelling markers factors and densitometric variables were not significantly related. Age, weight and pubertal stage were significantly related to bone mass.

**Discussion:**

Our results highlights the insufficient increase in bone mass with age (probably due to excessive bone remodelling) and confirms the high prevalence of low bone mineral density in children with severe cerebral palsy. Possible preventive measures might include calcium + vitamin D supplementation and the systematic management of underweight and delayed puberty. Bone remodelling markers might be of value for follow-up.

## Introduction

Cerebral palsy (CP) results from brain injuries sustained during the early stages of development (1). The condition affects approximately 2.5 children per 1,000 births (2). CP can be responsible for many motor, sensorial and cognitive impairments of varying degrees of severity, ranging from mild to multiple disabilities (3). It has been reported that 77% of non-walking children with CP have a low bone mineral density (BMD) and an elevated fracture risk (about 4% per year), particularly at the vertebrae and the distal femur (4). Limited mobility and feeding difficulties are often the main risk factors. Due to the frequent presence of comorbidities (such as epilepsy, orthopaedic disorders, and respiratory complications), bone health is often overlooked in children with CP. In fact, bone fragility and fracture can be responsible for pain, functional limitations (preventing pain when sitting), increased medication use and, in some cases, reduced life expectancy (5, 6). There are few literature data on these factors, which partly explains the delays in diagnosis, prevention and therapy observed in routine clinical practice. Furthermore, the evaluation of bone health in children with severe CP is challenging; this is especially the case for densitometric evaluations of the proximal femur and lumbar spine (LS). These two anatomical sites are difficult to interpret because of the high prevalence of scoliosis and hip dysplasia in this population (7, 8). For these reasons, we decided to assess the bone health of children with severe CP [in accordance with the 2019 guidelines on paediatric densitometry issued of the International Society for Clinical Densitometry (ISCD)] (9). We therefore assessed the BMD and bone mineral content (BMC) of the total body less head (TBLH) and the LS. The study's primary objective was to describe the children's densitometric variables. The secondary objectives were to determine whether any of the clinical and laboratory variables were associated with densitometric variables in this population and to identify risk factors for a low BMD.

## Method

### Study design and participants

We conducted an observational, retrospective, single-centre study in the paediatric physical medicine and rehabilitation unit at Amiens University Hospital (Amiens, France). The study database was registered with the French National Data Protection Commission [*Commission nationale de l'informatique et des libertés* (Paris, France); registration reference: PI2021_843_0036]. All non-walking children [Gross Motor Functional Classification System (GMFCS) levels IV and V (10)], with CP aged between 6 and 18 with a complete bone fragility assessment, including a DXA and a biological sample, were eligible for inclusion in the study. The children's bone health was evaluated during a routine day hospital admission between January 2020 and December 2022, with the collection of clinical, laboratory and dual-energy x-ray absorptiometry (DXA) data. Children with a fracture in the previous 3 months, those having undergone orthopaedic surgery in the previous year, those with metal orthopaedic implants, those who had a previous antiresorptive treatment and those with additional causes of bone fragility were excluded from the analysis.

### Clinical data

All the clinical data were obtained from the hospital's electronic medical records after entry with DxCare© (version 7.7.9.7.7, Dedalus, Le Plessis-Robinson, France) software upon discharge from hospital. The clinical data included sex, age, height, weight, Z score for height and weight, body mass index, pubertal status according to Tanner's classification*,* fracture history [only clinically significant fractures as defined by the ISCD in 2019 (9)], feeding method (feeding tube, *per os*, mixed), calcium intake [estimated with the French-language Fardellone questionnaire (11) or from the calcium content of gastrostomy bags], and the serum level of 25-hydroxy vitamin D [25(OH)D]. The calcium intake was classified as being sufficient or deficient for age, according to the recommended dietary allowances for the French population. 25(OH)D status was determined as deficient (serum level <30 nmol/L), insufficient (30–50 nmol/L) or sufficient (>50 nmol/L) (12–14). The serum levels of albumin (38–54 g/L) and insulin-like growth factor 1 (IGF-1) were used as biomarkers of nutritional status (15). A history of treatment with antiresorptive and/or anticonvulsant drugs was also taken in account because of the latter's potential interference with bone metabolism.

### Densitometry

All DXA measurements were performed by experienced technicians, using a Horizon 1 densitometer (serial number 302550M - Hologic, Marlborough, MA). To limit the impact of scoliosis on the acquisition of lumbar spine measurements, the children were examined in a position that stretched the spine as possible. The BMD (g/cm^2^), the BMC (g) and the Z score for age were obtained for each site (TBLH and LS). A Z score less than −2 was considered to be low. The BMD was adjusted for age, sex and ethnicity, according to the ISCD guidelines (9).

### Laboratory variables

Blood levels of calcium (normal range: 2.2–2.6 mmol/L), phosphorus (normal range: 0.78–1.45 mmol/L), and parathyroid hormone (PTH; normal range: 18.5–88 pg/ml) and vitamin D were measured in routine practice. Serum samples were stored at −80°C prior to further analysis of bone markers. Bone alkaline phosphatase (BALP; normal range: 5.5–24.6 µg/L) and osteocalcin (OC; normal range: 6.5–42.3 ng/ml for girls and 4.6–65.4 ng/ml for boys) were measured as bone formation markers, using a Liaison® XL (Diasorin, Saluggia, Italy) and Liaison® BALP and OC kits, respectively. The serum level of type 1 collagen C-terminal telopeptide (CTX, ng/L) was assessed as a marker of bone resorption, using a CrossLaps® ELISA (Immunodiagnostic Systems, Pouilly en Auxois, France). CTX serum levels were interpreted with regard to the age-adjusted normative values published by Crofton et al. (16).

### Statistics

All statistical analyses were performed with SPSS software (version 25, IBM-17 avenue de l'Europe, 92,275 Colombes, France). Quantitative variables were expressed as the mean [standard deviation (SD)] and qualitative variables were presented as the number (percentage). Non-parametric comparisons were carried out for quantitative data. Given the number of children included, a non-parametric Spearman test was used in bivariate correlation analyses. Correlation analyses were performed on variables considered to be clinically relevant. The significance threshold was set to *p* < 0.05.

## Results

### Clinical data

Of the 29 children admitted to our unit for a bone health evaluation, three were excluded because of the presence of osteosynthesis material, two had incomplete DXA datasets, and four had blood samples that were insufficiently large for all the planned laboratory analyses. Hence, 19 children had complete datasets and were included in the final analysis. There were 11 boys and 8 girls, and the mean (SD) age was 12.74 (3.08) years. The children presented with abnormally low height and weight values for age: the mean Z score was −2.92 (1.79) for height and −3.75 (3.64) for weight. According to Tanner's classification, 8 children were pre-pubertal (stage I or II) and 11 were pubertal (stage III or higher). Ten of the 19 children had a feeding tube. All the children had a normal nutritional status, according to the IGF-1 and albumin levels. Only three children had a normal calcium intake for age. The 25-OH-D concentration was adequate in 13 children, the same who benefit from vitamin D supplementation (50,000 IU every 3 months). One child had secondary hyperparathyroidism associated with a profound vitamin D deficiency [PTH: 159.8 pg/ml; 25(OH)D: 20 nmol/L]. All of the children had normal serum calcium and phosphorus levels (Table 1).

**Table 1:** Children's characteristics.

Demographic characteristics	
Sex (males/females), N	11/8
Age (years), mean (SD)	12.74 (3.08)
GMFCS (IV/V)	7/12
Height (m), mean (SD)	1.34 (0.16)
Z score height, mean (SD)	−2.92 (1.79)
Weight (kg), M (SD)	30.23 (9,24)
Z score weight, mean (SD)	−3.75 (3.64)
BMI (kg/m^2^), mean (SD)	16.96 (4.66)
Tanner stage (1/2/3/4/5)	6/2/6/3/2
History of fractures (Yes/No)	0/19
Nutritional status	
Feeding method (feeding tube, per os, mixed)	7/10/2
Albumin (normal/abnormal)	19/0
IGF-1 (normal/abnormal)	19/0
Calcium intake (sufficient/insufficient)	3/16
Vitamin D supplementation (Yes/No)	13/6
Phosphorus/calcium variables	
25-OH-D3 status (sufficient/insufficient/deficiency)	13/2/4
Calcium (mmol/L), mean (SD)	2.36 (0.09)
Phosphorus (mmol/L), mean (SD)	1.33 (0.18)
PTH (normal/elevated)	18/1
Treatments	
Anticonvulsants (yes, no)	10/10

### Densitometry

The mean (SD) BMC^TBLH^ was 605.140 (219.144), which corresponded to a mean densitometric Z score of −4.8 (1.8). None of the children had a normal Z score for the BMC^TBLH^. The mean BMD^TBLH^ was 0.595 (0.118), which gave a mean Z score of −4.4 (1.76). One child had a BMD^TBLH^ Z score at or above −2 (−1.5). The mean BMC^LS^ was 23.722 g (9.979) but the BMC^LS^ Z score could not be measured. The mean BMD^LS^ was 0.593 (0.127), giving a mean Z score of −2.1 (1.97). Six children had a BMDLS Z score above −2 (ranging from −2 to 1.1). The mean fat mass was 9,328.668 (4,743.858), and the mean lean mass was 17,479.721 (6,535.438). The absolute value of densitometric parameters was not different between GMFCS IV & V children which was not different while the densitometric Z score for BMD and BMC was significantly lower in GMFCS V children (Table 2).

**Table 2:** Densitometric variables.

Bone mineral content (BMC)	
BMC^TBLH^ (g), mean (SD)	605.140 (219.144)
Z score for BMC^TBLH^, mean (SD)	−4.8 (1.8)
Normal/ < −2 SD	19/0
BMC ^LS^ (g), mean (SD)	23.722 (9.979)
Bone mineral density (BMD)	
BMD^TBLH^ (g/cm2), mean (SD)	0.595 (0.118)
Z score for BMD^TBLH^, mean (SD)	−4.4 (1.76)
Normal/ < −2 SD	1/18
BMD^LS^ (g/cm2), mean (SD)	0.593 (0.127)
Z score for BMD^LS^, mean (SD)	−2.1 (1.97)
Normal/ < −2 SD	13/6
Soft tissues	
Fat mass (g), mean (SD)	9,328.668 (4,743.858)
Lean mass (g), mean (SD)	17,479.721 (6,535.438)

### Bone remodeling factors

Ten children had a serum CTX concentration above the age-normal value. The mean value was 1,036.77 ng/L (517.47). Most children had a serum BALP concentration above our laboratory's normative value; the children's mean value was 51.32 µg/L (29.38). The mean OC concentration was 59.53 ng/ml (33.44), and 11 of the 19 children had an elevated value (Table 3).

**Table 3:** Serum levels of specific bone remodelling factors.

Bone resorption	
CTX (ng/L), mean (SD)	1,036.77 (517.47)
Normal/elevated, *n*	9/10
Bone formation	
BALP (µg/L), mean (SD)	51.32 (29.38)
Normal/elevated, *n*	2/17
OC (ng/ml), mean (SD)	59.53 (33.44)
Normal/elevated, *n*	8/11

### Correlations between clinical, laboratory and densitometric variables

We observed a positive correlation between age and the absolute values of BMC^TBLH^, BMD^TBLH^, BMC^LS^ (*p* < 0.01) and lean mass (*p* < 0.05). Conversely, the oldest children had a significantly lower Z BMC^TBLH^ and Z BMD^LS^, although no significant correlation was found for Z BMD^TBLH^. Age was associated with lean mass but not with fat mass. Weight was positively correlated with all the densitometric variables (*p* < 0.01 for BMC-BMD^TBLH^, and *p* < 0.05 for BMC^LS^) other than BMD^LS^ and was only correlated with the Z BMD^TBLH^ (*p* < 0.01). The children's bodyweight was significantly related to soft tissue mass. Pubertal status was correlated with lean mass and with densitometric variables other than the BMD^LS^. No significant relationships between serum bone remodelling factors and densitometric variables were found. The concentration of the resorption marker CTX was negatively associated with age and the Tanner stage. Remodelling markers were strongly correlated with each other, with the exception of CTX vs. BALP (Table 4).

**Table 4:** Correlations between clinical, laboratory and densitometric variables.

	Age	Z score for height	Z score for weight	Weight	Tanner stage	CTX level	BALP level	OC level
Densitometric data								
BMC^TBLH^	**0**.588**	0,632	0.067	**0**.605**	**0**.579**	−0.389	0.034	−0.110
BMD^TBLH^	**0**.705**	0.113	−0.037	**0**.661**	**0**.631**	−0.316	−0.251	−0.267
BMC^LS^	**0**.629**	−0.121	−0.121	**0**.489*	**0**.637**	−0.386	−0.067	−0.170
BMD^LS^	0.427	−0.005	−0.005	0.440	0.440	−0.381	−0.069	−0.201
Z score BMC^TBLH^	**−0**.501*	0.368	**0**.774**	0.414	−0.253	−0.009	0.222	0.240
Z score BMD^TBLH^	−0.284	0.422	**0**.635**	**0**.527**	−0.186	0.098	−0.107	0.127
Z score BMD^LS^	**−0**.591**	0.345	**0**.729**	0.440	−0.416	0.264	0.147	0.406
Soft tissues								
Fat mass	−0.051	0.333	**0**.600**	**0**.703**	0.135	−0.177	−0.195	0.381
Lean mass	**0**.557*	0.228	0.175	**0**.805**	**0**.623**	−0.349	−0.075	−0.075
Bone marker levels								
CTX	**−0**.494*	0.010	0.147	−0.265	**−**0.261	–	−0.115	**0**.626**
BALP	−0.263	−0.093	0.232	−0.147	−0.186	−0.115	–	**0**.416*
OC	**−0**.476*	−0.218	0.207	−0.157	−0.339	**0**.626**	**0**.416*	–

Bold characters identify statistically significant results.

* *p* < 0.05.

** *p* < 0.01.

## Discussion

The results of our study [based on the ISCD's latest methodological guidelines on paediatric densitometry (9)] confirmed the high prevalence of low BMD in non-walking children with CP (GMFCS levels IV and V): none of the children had a normal BMC^TBLH^ Z score, and only one had a BMD^TBLH^ Z score greater than −2.

According to the reference curves and our correlation analysis, the children's bone mass increased insufficiently with age – as evidenced by the low Z scores. One can reasonably hypothesize that the early-onset osteoporosis observed in adults with CP starts in childhood. Our observation highlights the value of implementing early strategies to promote bone health in children with severe CP because the potential consequence is low peak bone mass, which increases the risk of adult osteoporosis (17).

Weight appears to be one of the most important clinical factors related to the bone mass in children with non-walking CP. Indeed, absolute weight is related to densitometric data, and underweight is linked to a low densitometric Z score. Paradoxically, the children's nutritional status was normal; how can this be explained, considering that weight is related to food intake, and that food insufficiency is related to weak bone mass (18)? Since low weight appears to lead to short stature for age (19), it is likely that the biomarkers assayed here did not fully reflect the children's nutritional status. The clinical data collected also showed that 13 children (70%) had adequate serum vitamin D levels. However, only one child had an adequate calcium intake for his age. Given the low UV exposure (20) and frequent use of anti-epileptic drugs observed in children with multiple disabilities (21), our findings emphasize the need to consider calcium supplementation as well as conventional vitamin D supplementation; the combination of the two contributes to better bone health (22). As pubertal status is related to bone mass in children with CP, physicians should systematically screen for delayed puberty due to hypogonadism (23, 24). We suggest that weight management, implementation of appropriate vitamin + calcium supplementation, and screening for and then treatment of delayed puberty are essential for promoting bone health in children with severe CP and should be incorporated into primary prevention strategies.

We used a biochemical approach to study bone metabolism. None of the associations between densitometric variables and remodelling markers were statistically significant. However, half of the children had elevated serum CTX levels (16). Considering the positive correlation between remodelling factors in this study, excessive bone remodelling might explain the low DXA results. Given the difficulties in performing DXA in these children, assay of remodelling factors can usefully complement bone assessments during patient follow-up.

Although we chose to evaluate children with the most severe motor impairments, no fracture was documented - that is well below the literature data (ranging from 12% to 23%) (4). Severe motor impairment is typically associated with severe cognitive impairment (including language), as found in all children in the present study. In addition to asymptomatic fractures [as is sometimes the case in typical osteoporosis (25)], the children's motor impairments and poor speech skills might have masked a number of undiagnosed fractures. As is also the case for healthy children, the present results for children with CP did not enable us to conclude that densitometric variables are related to the fracture risk. We suggest that fractures should be systematically screened for with target zone radiography or more specific techniques, such as vertebral fracture assessment; this might optimize therapeutic management and preventing further prevalent fractures.

The present study had several limitations. Firstly, the small size of the study population might have limited the power of our statistical analyses. Secondly, the study's cross-sectional design prevented us from determining how the densitometric and laboratory variables changed over time. Hence, our results need to be confirmed in a longitudinal study, which might also provide more detailed information on the physiological mechanisms underlying the low observed BMD in children with CP and identify the most practical, comprehensive, therapeutic strategies in this context.

However, our study had several strengths. Firstly, it was based on the exhaustive collection of clinical data. Secondly, we compared the clinical data with laboratory and densitometric data, in accordance with the ISCD's latest guidelines; in fact, very few studies of children with severe CP have been carried out in this way. Together, our results confirmed the absolute need for very early interventions that promote bone health in these children.

## Conclusion

Our results confirmed that children with severe CP are likely to have poor bone mineralization with regard to their weight and pubertal stage. Furthermore, the results suggest that growth worsens the lack of mineralization (compared with healthy children of the same age) and confirmed the absolute need for preventive action. Although the levels of bone turnover markers were not related to densitometric variables, it appears that high bone remodelling favoured bone loss. Systematic calcium supplementation (along with vitamin D3 supplementation), weight management, and monitoring of pubertal development might be useful ways of improving overall bone health in children with CP. Given the heterogeneity of patient profiles in CP, longitudinal studies are needed to confirm these observations and to determine the values of bone remodelling biomarker assays in routine clinical practice.

## Data Availability

The original contributions presented in the study are included in the article, further inquiries can be directed to the corresponding author.
